# Review: Structure-Activity Relationship of Antimicrobial Peptoids

**DOI:** 10.3390/pharmaceutics15051506

**Published:** 2023-05-15

**Authors:** Priscilla L. Nyembe, Thandokuhle Ntombela, Maya M. Makatini

**Affiliations:** Molecular Sciences Institute, School of Chemistry, University of the Witwatersrand, Johannesburg 2050, South Africa

**Keywords:** antimicrobial peptides, antimicrobial peptoids, chain length, cationic, hydrophobicity, cyclization, amphiphilicity

## Abstract

Due to their broad-spectrum activity against Gram-negative and Gram-positive bacteria, natural antimicrobial peptides (AMPs) and their synthetic analogs have emerged as prospective therapies for treating illnesses brought on by multi-drug resistant pathogens. To overcome the limitations of AMPs, such as protease degradation, oligo-N-substituted glycines (peptoids) are a promising alternative. Despite having the same backbone atom sequence as natural peptides, peptoid structures are more stable because, unlike AMP, their functional side chains are attached to the backbone nitrogen (N)-atom rather than the alpha carbon atom. As a result, peptoid structures are less susceptible to proteolysis and enzymatic degradation. The advantages of AMPs, such as hydrophobicity, cationic character, and amphipathicity, are mimicked by peptoids. Furthermore, structure-activity relationship studies (SAR) have shown that tuning the structure of peptoids is a crucial step in developing effective antimicrobials.

## 1. Introduction

The rising concern of antibiotic-resistant microorganisms has prompted a renewed interest in antimicrobial peptides (AMPs), which have a good application potential in the field of agriculture, medicine, aquaculture, and food [1]. AMPs are defined as a group of antimicrobial agents capable of fighting infectious diseases in living organisms by killing or inhibiting pathogens [2,3,4]. They exhibit a wide range of activity against diverse microorganisms. Because of their unknown mechanism of action, most bacteria rarely develop resistance against them [5]. According to the antimicrobial peptide database (APD31) revised on 24 August 2020, 3240 AMPs have been recorded [1]. The common features observed in different types of AMPs are as follows: They are short, with an average of 33.26 amino acids, amphipathic (having both hydrophobic and hydrophilic regions), and just about every AMP is cationic (+2 to +9 net charge; average 3.32). Moreover, there are several anionic AMPs, which consist of amino acids that are acidic, such as aspartic acid and glutamic acid [1,6].

**Figure 1:**
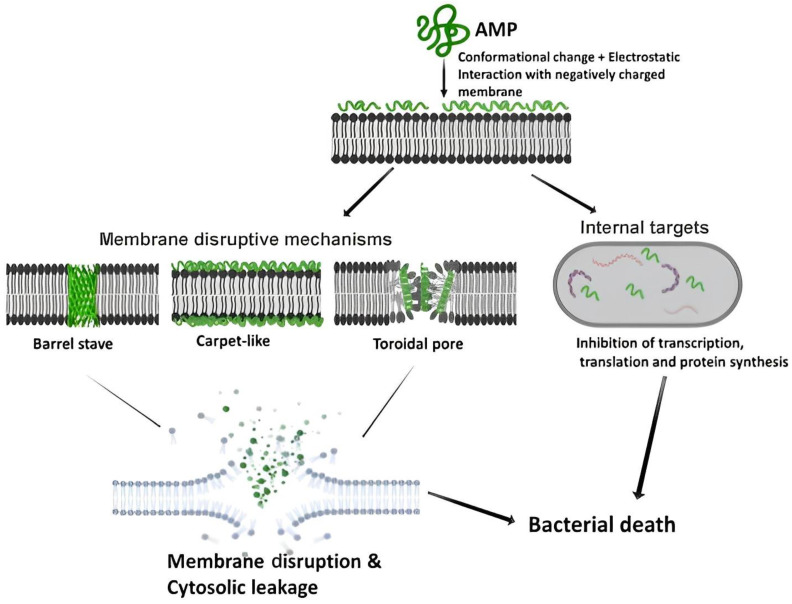
The AMPs membrane disruptive and non-membrane disruptive mechanisms for killing bacteria [10]. Reprinted with permission from [10], published by Zoological Research, 2019.

The hydrophobic interactions, together with the non-polar lipid acyl chains (AMPs’ amphiphilic structure), aid the peptide’s initial electrostatic connection with the anionic bacterial membrane lipids [7]. The cationic character of AMPs makes them more selective towards the negatively charged cytomembrane of the bacteria over the less formally negatively charged membrane of eukaryotes [8,9]. The AMPs’ broad-spectrum success can be attributed to the comprehensive membrane interactions they employ to eliminate foreign bodies in the host. AMPs mainly act by breaking down or disrupting the bacterial cell membrane, while some act via the non-membrane disruptive mechanism (Figure 1). The class of AMPs that kill through the membrane disruptive mechanism mostly do so by permeating the cytomembrane, which results in the release of intracellular material. For the three proposed membrane disruptive mechanisms, namely the Barrel stave, toroidal pore, and carpet-like model, the AMP molecules first accumulate and organize themselves parallel to the cytomembrane surface. Followed by the electrostatic attraction between the bacterial cell envelope that is slightly negative and the partly positive amino acid side chains in the AMP [10].

The folded peptide will position itself such that the charge centers are situated at the front of the membrane, then, weak hydrophobic interactions are established [9]. The non-membrane disruptive AMPs can exert intracellular effects like obstructing the synthesis of the cell wall, protein, and nucleic acid by passing through the cell membrane’s lipid bilayer without permeabilization [10].

Host-defense AMPs mainly targeting the bacterial membrane have been previously studied as new antibiotic agents. Despite their effectiveness, AMPs have various drawbacks. They are easily hydrolyzed by proteases (in vivo protease degradation), which lowers their bioavailability, and their high cost of synthesis limits their production. Moreover, they are unstable at certain pHs and have possible immunogenicity and/or systemic toxicity [7,11,12,13,14].

These limitations pose a challenge for converting AMPs from the bench to the market as therapeutic agents for infections that have developed resistance to their respective drugs. Ideally, AMP should possess the following qualities: (1) High potency against microbes, (2) low toxicity towards mammalian membranes, (3) stable against protease degradation and the environment, (4) accessible and affordable cost of manufacturing [6]. The design of AMPs that exhibit the desired effect has attracted much attention in research. Much effort has been made to exploit and amplify the features present in naturally occurring AMPs. Therefore, several non-natural mimics of AMPs with better bioavailability and biostability have been invented and synthesized, thus likely enhancing pharmaceutical suitability. 

**Figure 2:**
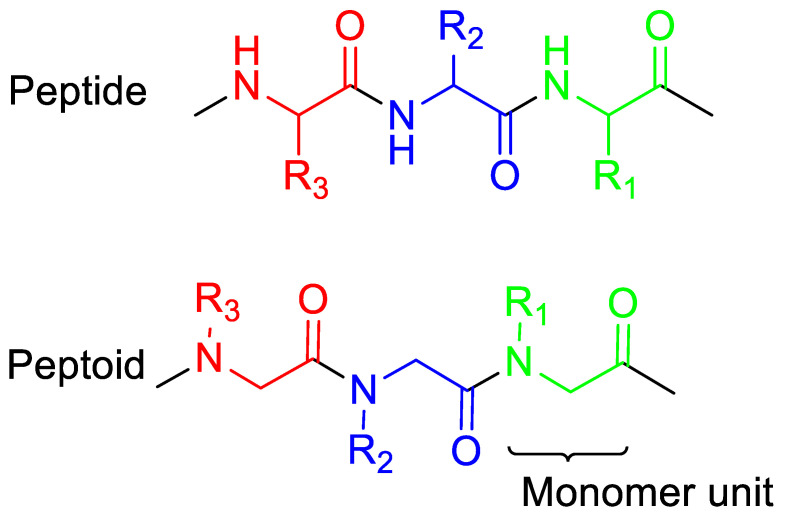
The structures of peptoids vs. peptides. Peptoids are synthetic analogs of peptides in which the side chains (R_1_, R_2_, R_3_) are attached to the nitrogen atoms of the backbone rather than the α-carbons as in peptides.

Oligo-N-substituted glycines (peptoids) are a favorable substitute for AMP. Peptoids have similar backbone atom sequences as AMPs, however, they are less vulnerable to enzymatic and protease breakdown, hence, they have a higher potential to be utilized as pharmaceuticals and in biomaterials. Unlike AMPs, peptoid functional side chains are anchored to the nitrogen (N)-atom rather than the alpha-carbon (Figure 2) [15]. This structure makes peptoids more stable because no investigated protease can recognize and degrade them. Furthermore, there are more options for primary amines with different side chains, which can be included in the peptoid chain through the submonomer synthetic strategy [15,16,17,18,19]. Due to the peptides’ prospective toxicity being a great barrier that limits their use clinically, peptoids’ low cytotoxicity respective to the AMP pexiganan further strengthens their therapeutic potential [16].

**Figure 3:**
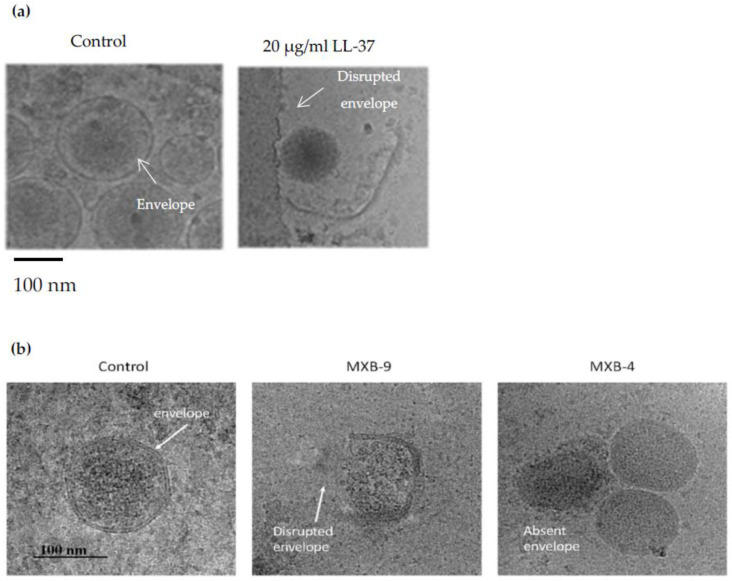
Peptoids appear to act by disrupting the microbial membranes just as AMPs. Shown above are the Cryo-EM results from the studies conducted by Brice, D.C et al. (2018) [22] and Diamond, G et al. (2021) [23] for AMP and peptoids, respectively (**a**) Shows the disruption of Kaposi’s Sarcoma Herpes Virus (KSHV) envelope by the AMP called LL-37 [22]. Similarly, (**b**) when SARS-CoV-2 was treated with two active peptoids (MXB-4 and MXB-9), several slightly disrupted membranes (middle) were observed and appeared to be nucleocapsids without envelopes. These structures were not observed in the control samples, proposing that the peptoids act via the same membrane-disruptive mechanism on MXB-4 and MXB-9 virus types [23]. Reprinted with permission from [23], published by Pharmaceuticals, 2021.

The bacterial membrane is more permeable to peptoids than peptides [19,20] and similar to AMPs, peptoids act by either disrupting the membrane of the bacteria (Figure 3) or by targeting intracellular targets like bacterial DNA [15]. Recent studies have shown the antimicrobial properties of peptoids, such as a cationic and amphipathic dodecamer peptoid 1 [21], which displayed biological activity against a wide range of bacteria and fungi. Other studies have suggested that antimicrobial peptoids act through the same mechanism as AMPs due to the retained antimicrobial activity when peptides are translated to peptoids. The acquired AMP knowledge can be applied in developing peptoid antibiotics because they mimic AMPs structurally and functionally.

**Figure 4:**
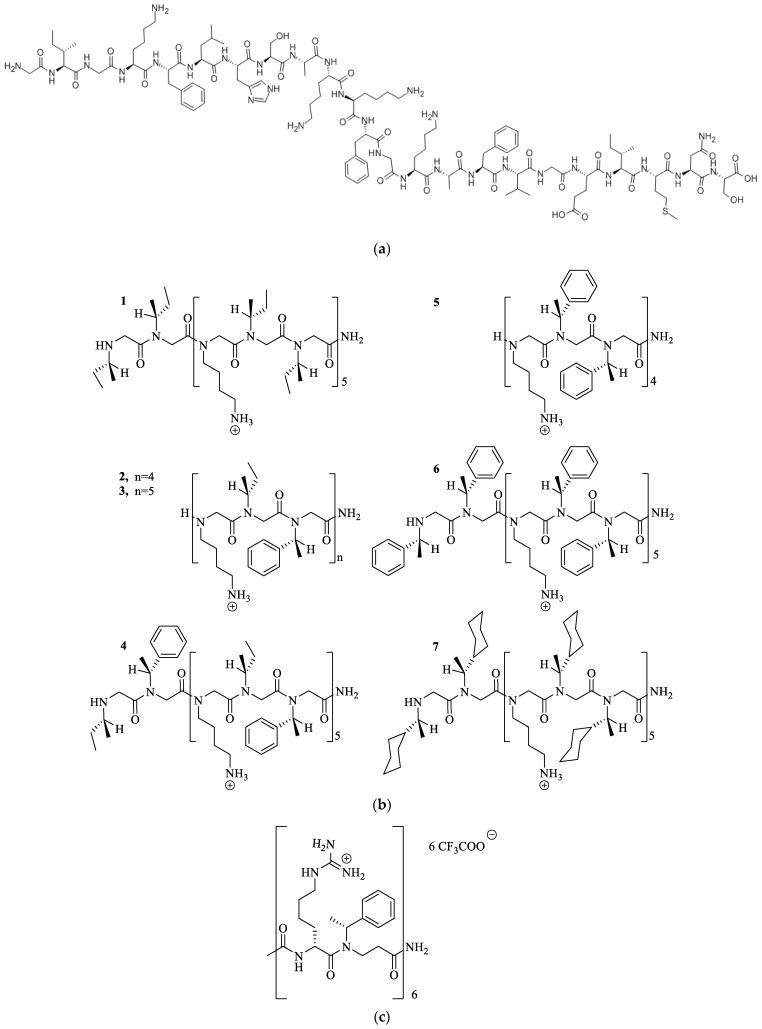
(**a**) The structure of magainin-2 amide [24] and (**b**) the peptoid mimetics (1–7) of magainin-2 amide showed antibacterial effects with poor hemolytic activities. Reprinted with permission from [24], published by American Chemical Society, 2003 [24] (**c**) The structure of alpha/beta peptoid chimeras demonstrated selective toxicity towards bacterial cells and displayed protease stability [24].

Barron et al. (2003) were the first group to demonstrate that the peptoid mimicking the structure of an AMP called magainin-2 amide (Figure 4a,b) displayed antibacterial effects with poor hemolytic activities [24]. This group recently assembled antimicrobial peptoid oligomers that mimicked the structure of the AMP called pexiganan. These peptoids showed broad-spectrum activity (MIC 0.88–7.4 mg/L) and low mammalian cytotoxicity [25,26,27]. Jaroszewski et al. (2007) [27] synthesized alpha-peptide/beta-peptoid chimeras (Figure 4b) that were selectively toxic to bacterial cells and displayed protease stability. These chimeras showed good antibacterial activity against *Bacillus subtilis* and *Escherichia coli* (MICs 7.5–13.5 mg/L) [24,25].

Studies on the structural-activity relationship (SAR) have shown how important it is to modify the structure of peptoids when creating effective antimicrobials. Chongsiriwatana et al. (2008) [25] studied helical peptoids that show activity against various microorganisms. They demonstrated that the overall charge and average hydrophobicity are key for the antimicrobial activities displayed by the peptoids, while high hydrophobicity and amphipathicity resulted in hemolysis. Moreover, Mojsoka et al. (2020) looked at how hydrophobicity affects the activity of peptoids by designing a collection of short, linear, hydrophobic, and cationic peptoids with modifications while keeping the charge constant. They concluded that high hydrophobicity improved the potency against *Staphylococcus aureus* in vitro [15].

Many studies have frequently mentioned five aspects that must be considered during the logical design of antimicrobial peptoids, including the length of the chain, hydrophobicity, secondary structure, net charge, and amphiphilicity. In this review, we highlight how different structural properties of the peptoids influence their biological activity against antimicrobials. 

## 2. Main Chain Length

Recently, linear AMPs have been designed to be ever-short for synthetic and pharmacological reasons [28,29,30,31,32,33]. Synthetic oligomers, particularly those that target the bacterial membrane, have notable potential for clinical development [34]. A lot of membrane-active natural antimicrobial oligomers comprise over 10 monomeric residues, and their molecular weights are bigger than 1000 Daltons [34,35]. The downside of such compounds is that they may face inherent disadvantages concerning pharmacological development and are usually restricted to being applied topically in the clinic. Chain length also influences the cost of production and hence the market price. To circumvent the issue of the high cost of synthesizing AMP, Svendsen et al. (2008) worked on developing short AMPs, which consisted of only three amino acid residues. These short cationic AMPs showed a wide-ranging antimicrobial activity against Gram-positive (MICs of 2.5 mg/L, including methicillin-resistant *S. aureus* (MRSA)) and Gram-negative bacteria (MIC of 5.0 mg/L, including *P. aeruginosa*) [11]. Furthermore, these peptides were selective against bacterial cells compared to mammalian cells [11,25]. It is crucial to investigate how the length influences the biological activity of the peptoids.

### The Effects of Main Chain Length on Antimicrobial Activity

Huang et al. (2012) studied the influence of molecular size on antimicrobial activity by analyzing oligomers of six, eight, and ten subunits [7]. They noticed a correlation between the antibacterial activity of linear and cyclic oligomers and the length of the chains. The observed decline in MIC value as the oligomer length increases from 6 to 10 subunits indicates that, in general, longer chain lengths improve the antimicrobial activity for both the cyclic and linear series. Their linear decamer, the largest out of the linear sequences, showed the highest potency regarding antimicrobial activity (MIC of *E. coli* = 31.3 µg·mL^−1^). Comparably for the cyclic series, their decamer with the largest macrocycle showed the highest antimicrobial activity potency (MIC of *E. coli* = 7.8 µg·mL^−1^) [7]. 

Mojsoska et al. (2015) studied the implication of main chain length on the potency displayed by their peptoids by truncating two of their maternal peptoids that had similar structural composition (besides the residue on position 9) from the C-terminal end. They claimed that the shortened peptoids had better antibacterial action against *Staphylococcus aureus (S.aureus)* and reduced retention time. They also deleted the 9th residue from peptoid 3 (Table 1) and observed no change in their activity. These findings demonstrate that reducing the peptoid chain by one monomer can increase antimicrobial activity. Deleting the first monomer at the C-terminal can also result in higher eukaryotic toxicity, as observed on their peptoid 3 [12]. Comparably, an increase in antimicrobial activity and a decline in hemolytic property was seen when a leucine residue was deleted from the C-terminus of the peptide Ac-LKLLKKLL-KKLKKLLKKL-NH_2_ [36]. It can, therefore, be concluded that deleting one small residue that is hydrophobic from the carboxyl end decreases the hydrophobic character of the peptoid that results and increases the effectiveness of peptoids 7 and 3 as antibacterial agents against *E. coli* and *S. aureus* (Table 1) [36].

The length effect on peptoids’ antimicrobial activity and selectivity can mainly be accredited to the increasing hydrophobicity (higher reverse phase-HPLC retention time), which increases correlatively with chain length. However, the antimicrobial activity is maximized at a specific ideal hydrophobicity, and adding more hydrophobicity will only increase hemolytic activity.

## 3. Cationic Peptoids

The side chains that are positively charged within the peptoid sequence provide some selectivity between the zwitterionic plasma membrane of mammals and the more anionic plasmalemma of prokaryotes [37].

### 3.1. Lysine or Arginine Type Side Chains

**Figure 5:**
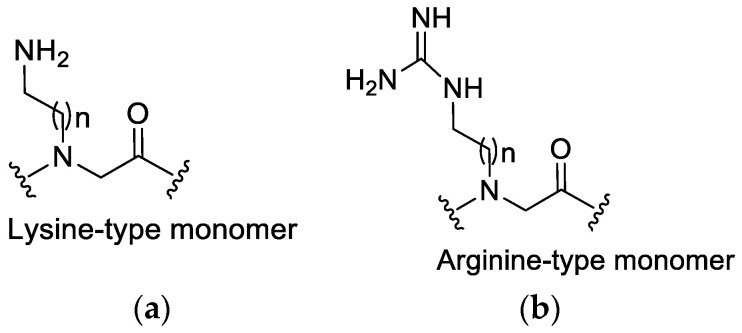
The structure of (**a**) Lysine-type monomer and (**b**) Arginine-type monomer.

The positively charged side chains in peptoids originate from lysine- or arginine-type monomers (Figure 5) in the chain. Some groups have researched the distinction between the incorporation of arginine- and lysine-type side chains for cellular absorption and demonstrated that there is faster cellular uptake for peptoids that consist of guanidine than their amino analogs [37]. Moreover, arginine-type monomers have been reported to possibly improve biological activity when incorporated into the sequence, although this may also increase toxicity toward mammalian cells [37,38]. This indicates the need for more research focusing on peptoids that consist of both the arginine- or lysine-type monomers to establish a balance between activity and toxicity. Most peptoids that have been studied only contain either all guanido functionalized (arginine-type) monomers or amino-functionalized (Lysine-type) monomers. This was due to the absence of a synthetic strategy to prepare mixed cationic peptoids [37]. Until Bolt and Cobb (2016) presented an efficient synthetic route that can be followed to synthesize linear and cyclic novel cationic peptoids that consist of both lysine-type and arginine-type monomers within the same sequence [37].

It is worth noting that, in the study by Biljana Mojsoska’s et al. (2020) [15], a decrease in antimicrobial activity was observed when arginine monomers within the structure of the peptide were completely replaced with lysine. This substitution might be the reason behind the peptoid mimics displaying lower antibacterial activity. Previous studies have proven that peptides having the lysine residues replaced with arginine residues showed a decrease in antimicrobial activity, which may be attributed to lower affinity to the membrane [21,39]. Furthermore, Amirkhanov et al. (2021) showed that replacing arginine residues with lysine or histidine residues in their synthetic antimicrobial peptides (SAMPs) fundamentally reduced their antibacterial properties in succession: **P1**-Arg > **P2**-Lys ≫≫ **P3**-His [40].

### 3.2. The Effect of Cationic Side Chains on Antimicrobial Activity

**Figure 6:**
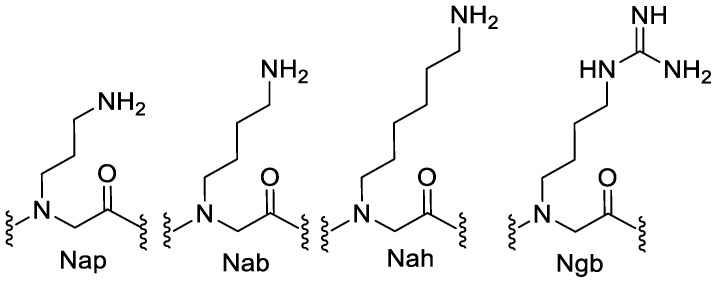
The structures of cationic monomers used in the study. Nap: N-(3-aminopropyl)glycine, Nab: N-(4-aminobutyl)glycine, Nah: N-(6-aminohexyl)glycine, and Ngb: N-(4-guanidinobutyl)glycine.

Studies that analyzed peptoids containing cationic residues with varying alkylamino and guanidino functional groups demonstrated that N-acetylated linear hexamers consisting of N-(4-aminobutyl)glycine (Nab) or N-(6-aminohexyl)glycine (Nah) (Figure 6) within their sequences were not active (MIC > 500 µg.mL^−1^) against all bacteria that were tested. On the other hand, moderate antimicrobial activity was observed on N-(3-aminopropyl)glycine (Nap)- and N-(4-guanidinobutyl)glycine (Ngb)-containing hexamers (MIC: 125–250 µg·mL^−1^). Among various cationic molecules tested in the same study, no consistent connection was observed between different chain lengths of the amino alkyl side chain or the nature of guanidino vs. amino functionality and the antimicrobial activity [7].

**Figure 7:**
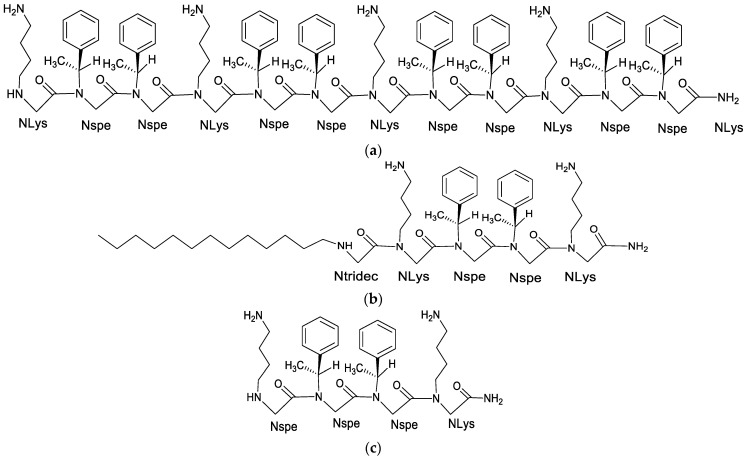
The 2D structures of (**a**) Peptoid 1, (**b**) Peptoid 1-C13_4mer_, a four residue long peptoid 13-carbon aliphatic tail attached to the N-terminus, and (**c**) the unalkylated peptoid 1_mer_.

A study focusing on anti-tubercular peptoids used a cationic, four-residue long peptoid 1-C13_4mer_ (Figure 7) to demonstrate the potency of cationic, biomimetic peptoids against a group of infectious bacteria that cause tuberculosis. The hydrophobic tail of peptoid 1-C13_4mer_, which is a 13-carbon aliphatic tail attached to the N-terminus, imparts it with considerable surfactant character—as a monomer. This tail is ideal because it is more likely to have a stronger and disruptive interaction with the hydrophobic, phospholipid bilayer of *Mycobacterium tuberculosis*, thus allowing the peptoid to penetrate the membrane of the bacteria more efficiently when compared to the unalkylated peptoid 1_4mer_. Cationic surfactants are generally toxic to the cells of mammals and bacteria, however, in the case of peptoid-C13_4mer_, its strong self-association enforced by the hydrophobic C13 tail should protect the outer membranes of the anionic (or hydrophobic, in the case of *Mtb*) macrophages that are not significantly anionic in contrast with bacterial membrane [21]. The *Mtb* has a cell membrane different from normal Gram-positive and Gram-negative bacteria. The *Mtb* cell membrane is made up of hydrophobic layers of mycolic acid, and this acyclic, hydrophobic barrier lowers the penetration capability of anti-TB drugs towards their target site, which in part, explain the high *Mtb* resistance to the available antibiotics.

When NLys Monomers were substituted with glutamate-like Nglu by Nathaniel and Chongsiriwatana et al. (2008), the resulting zwitterionic peptoid had considerably lower activity against *B. subtilis* and was not active against *E. coli*. This could be caused by the lack of favorable electrostatic interactions with the anionic bacterial cell membranes, but this zwitterionic peptoid was moderately hemolytic. On the other hand, the fully anionic variant did not show antibacterial and hemolytic activity. In conclusion, antimicrobial peptoids are selectively active, given they are cationic and adequate but not extremely hydrophobic, compatible with what has been reported on selective AMPs [25].

## 4. Hydrophobicity

Antimicrobial molecules are driven by the hydrophobic interactions to migrate from the aqueous environment and into the cell membrane [41]. Natural peptides that are extremely hydrophobic display higher cytotoxicity. It is, therefore, crucial to balance the hydrophobic and polar content of the peptide in order to regulate the selectivity of AMPs and non-peptidic therapeutic agents [41,42,43].

### 4.1. Influence of Hydrophobicity on the Secondary Structure of Peptides

According to research done on linear cationic AMPs in model membrane bilayers using circular dichroism (CD) and attenuated total reflectance-Fourier transform infrared spectroscopy (ATR-FTIR), non-polar environments cause peptides with more hydrophobic surfaces to undergo conformational modifications that change their secondary structure from random coil to alpha-helical and from alpha-helix to beta-sheet [41,44]. They also have a higher chance of forming aggregates than their less hydrophobic analogs in such environments. This can be attributed to a charge compensatory effect. The binding of peptides to the anionic phospholipids on the membrane of the bacteria leads to the creation of a dehydrated environment that favors the formation of beta-strand aggregates [45,46]. Moreover, this behavior is perceived in the viral fusion peptides from HIV-1 gp41 modulated by the cholesterol level in the targeted membrane [47,48].

### 4.2. Effect of Hydrophobic Surface Area (SA) on Antimicrobial Activity

Structure-activity relationship (SAR) studies on AMPs have proposed a strong connection between net hydrophobicity and antimicrobial activity. This is slightly due to the powerful hydrophobic interconnections between peptides and the target plasmalemma, which is much stronger in cases such as mammalian membranes, where the membrane comprises zwitterionic lipids. Barron et al. (2003) also reported a similar study in which the hemolytic properties and antibacterial activity increased when the hydrophobic character of the peptoids that mimic AMPs was increased [14,30]. Mojsoska et al. (2015) studied how hydrophobicity affected the activity and cytotoxicity of peptoids by designing a short, cationic, hydrophobic, and linear peptoid library with modifications while keeping the charge constant. When tested in vitro against *Staphylococcus aureus*, peptoids with higher hydrophobicity showed improved potency, but not when tested against *Escherichia coli* or *Pseudomonas aeruginosa* [12].

#### 4.2.1. Phenyl Monomers

**Figure 8:**
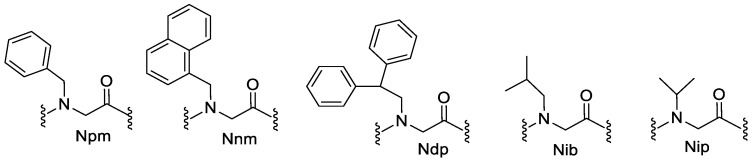
The structures of hydrophobic monomers. Npm: N-(phenylmethyl)glycine; Nnm: N-(1-naphthylmethyl)glycine; Ndp: N-(2,2-diphenylethyl)glycine; Nip: N-(isopropyl)glycine; Nib: N-(isobutyl)glycine.

Huang et al. (2012) studied how the hydrophobic surface area affects antimicrobial activity by conducting an experiment using three sets of oligomers which consisted of different phenyl residues of different levels of hydrophobic SA (Figure 8). The first set (L6 and C6) had the lowest hydrophobic SA in the group. The hydrophobic functional groups for this first set were three phenyl groups for each molecule. The second pair (L4 and C4) had greater hydrophobic SA than the first pair, which consisted of three naphthyl groups on each molecule as the hydrophobic functional groups. The last pair (L3 and C3) had the highest hydrophobic SA in the group, and six phenyl groups were incorporated as the hydrophobic functional groups per molecule. The antimicrobial data compiled from this study showed that the incorporation of Ndp residues into sequences had greater antimicrobial activity than those made up of Npm or Nnm residues. This observation proposes a compelling connection between antimicrobial activity and hydrophobic surface area. Furthermore, greater improvement of antimicrobial activity with increasing hydrophobic SA was much more noticeable in cyclic peptoids than those that were linear (Table 2) [7].

#### 4.2.2. Effects of Increasing Hydrophobicity by Nlys Substitution

**Figure 9:**
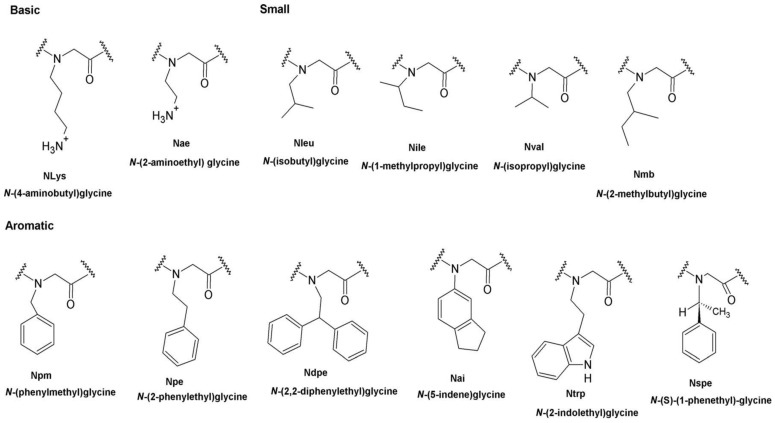
2D structures of peptoid monomers and their corresponding abbreviations and full names. Reprinted with permission from [12], published by AAC, 2015.

New peptidomimetics with enhanced antibacterial and hemolytic activities have been previously synthesized using lysine variants [49,50]. The Nlys residues (structure shown in Figure 9) contribute charge to the peptoids and are considered adequate for the first electrostatic interaction with bacteria, giving them superior affinity and, thus, anti-mycobacterial activity, unlike their respective hydrophilic analogs. 

A study was carried out to explore the implications that the length of a charged residue will have on the activity of the peptoid [12]. In that study, a shorter monomer, Nae, was replaced with Nlys, which led to minimal alteration to the retention time of the resulting peptoid. Similar observations were noticed for Nae substitution with Nlys, in which little influence on the shorter lysine version could be noticed in low dosage needed to lyse 10% of human red blood cells. Additionally, when this amount was decreased to 50% within the same sequence, the peptoid was optimized and showed better antibacterial activity and a decrease in toxicity [12].

#### 4.2.3. Effects of Increasing Hydrophobicity by Ntrp Substitution

Replacing Ntrp with monomers within the sequence can significantly impact the overall hydrophobicity profiles of the peptoids. In the study conducted by Mojsoska et al. (2015), where Ntrp was substituted with Nspe, the findings showed an increase in retention time on the resulting peptoid. However, a decline in antimicrobial activity by four-fold was observed for this peptoid against *S. aureus*. The antimicrobial activity did not improve when Ndpe was substituted with Ntrp [12]. From their results, this group concluded that where the monomers are positioned within the peptoid backbone could influence the observed behavior of the peptoid analogs in their study. They also suggested that extreme hydrophobicity may justify the observed decrease in antimicrobial activity for one of their peptoids (peptoid 16) [12], in which a strong peptoid-self association will hinder the peptoid from permeating across the bacterial cell wall. This has been proposed as a possible explanation for the substantial decline in the peptide antibacterial activity upon the increase in hydrophobicity [44].

#### 4.2.4. The Effect of Increasing Hydrophobicity by Single-Monomer Substitution

Hydrophobic amino acids that are small, like valine, isoleucine, and leucine, are frequently found in AMP structures in animals and bacteria [51]. The single-monomer change of leucine and isoleucine in the study by Mojsoska et al. (2015) resulted in a reduced antibacterial activity, and low toxicity was maintained when these peptoids were tested at the highest concentrations. The opposite effect was observed when only one Ntrp residue was substituted with an aromatic monomer that is bulkier, Ndpe. This exchange increased hydrophobicity, which significantly enhanced the potency of the peptoid against *S. aureus*, human red blood cells, and HeLa cells [12]. To further compare monomers with the same physiochemical traits but different in a single residue, they synthesized peptoids that contain bulkier residue, Ndpe, instead of Ntrp. When tested against *S. aureus*, *P. aeruginosa*, and *E. coli* strains, the resulting peptoids demonstrated similar antimicrobial activities with high hemolytic activity, resulting in 10% hemolysis at lower concentrations [12].

**Figure 10:**
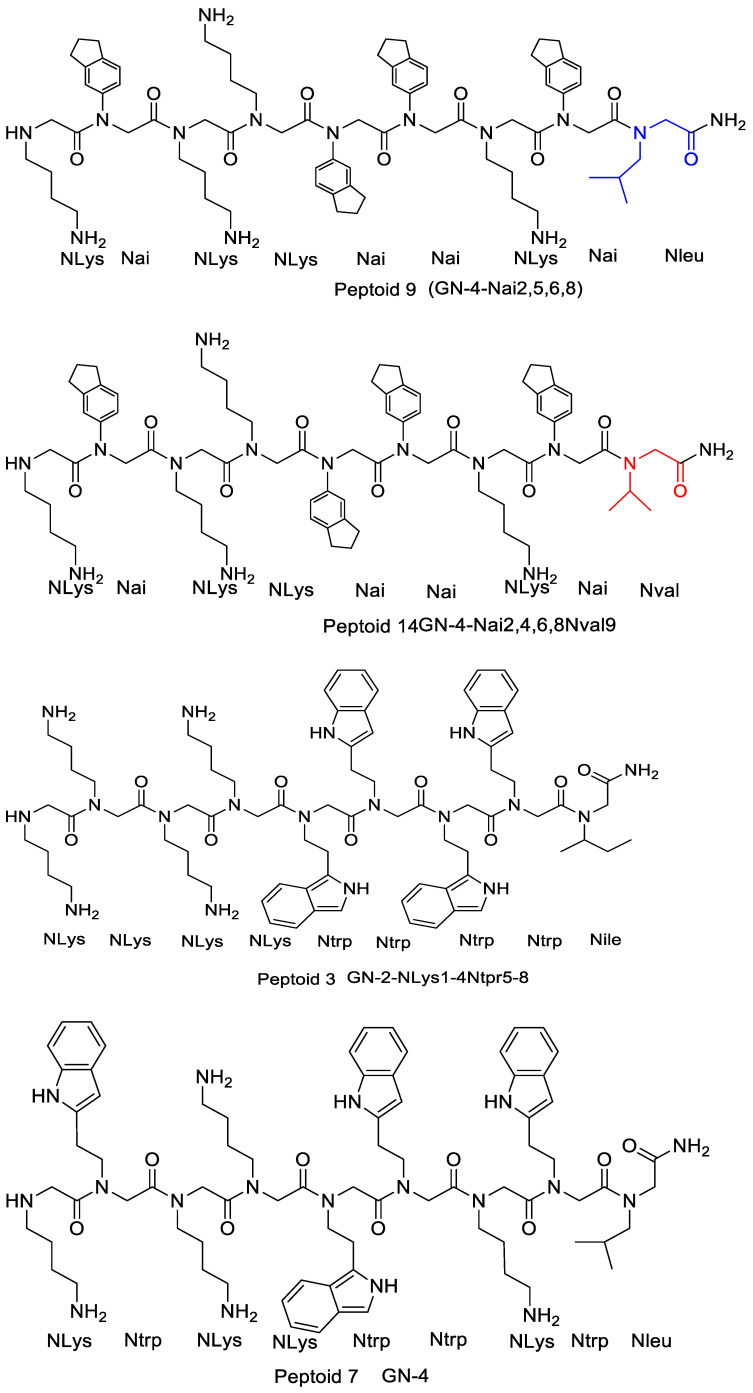
The chemical structures of peptoid 3, peptoid 9, peptoid 7, and peptoid 14.

In the investigation of two peptoids (peptoids 9 and 14 in Figure 10) whose Nval and Nleu monomer compositions varied, and which had Nai residue across the backbone rather than Ntrp, the much hydrophobic peptoid 9 showed improved antibacterial activity against the MRSA and the *E. coli* clinical isolate demonstrating extended spectrum -lactamases (ESBL) by two-fold [12]. Despite a direct correlation between hydrophobicity and potency observed against Gram-positive *S. aureus*, this group determined that high hydrophobicity in peptoids does not consistently seem as highly powerful against Gram-negative *E. coli* and *P. aeruginosa*. Comparatively, increasing the antibacterial activity of the resultant peptide and decreasing its hemolytic property was achieved by deleting the leucine residue from the carboxylic end of the peptide with the sequence Ac-LKLLKKLL-KKLKKLLKKL-NH_2_. 

Therefore, deleting one small hydrophobic residue from the carboxylic end decreases the resulting peptoids’ hydrophobic character that ultimately enhanced the antimicrobial activity observed for peptoids 7 and 3 (Table 1, Figure 10) against *S. aureus* and *E. coli* [12].

## 5. Amphiphilicity

Peptidomimetics that contain hydrophobic and charged groups in their sequence segregate into amphiphilic structures vital for interactions with the membrane of the bacteria [4]. Antimicrobial peptoids are usually created to be amphipathic with a combination of hydrophobic and positively charged residues. As the introduction states, this structural feature facilitates the first electrostatic interaction between the bacterial phospholipid head groups and the AMPs and the hydrophobic interactions with the non-polar lipid acyl chains. It also makes the peptoid selective for the cells of the bacteria, lowers toxicity to the cells of mammals, and enhances their activity as molecular transporters.

### Effects of Amphiphilicity by Sequence Rearrangement

**Figure 11:**
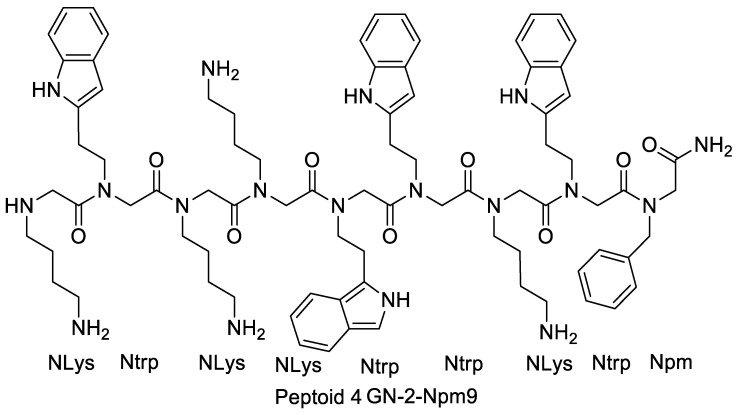

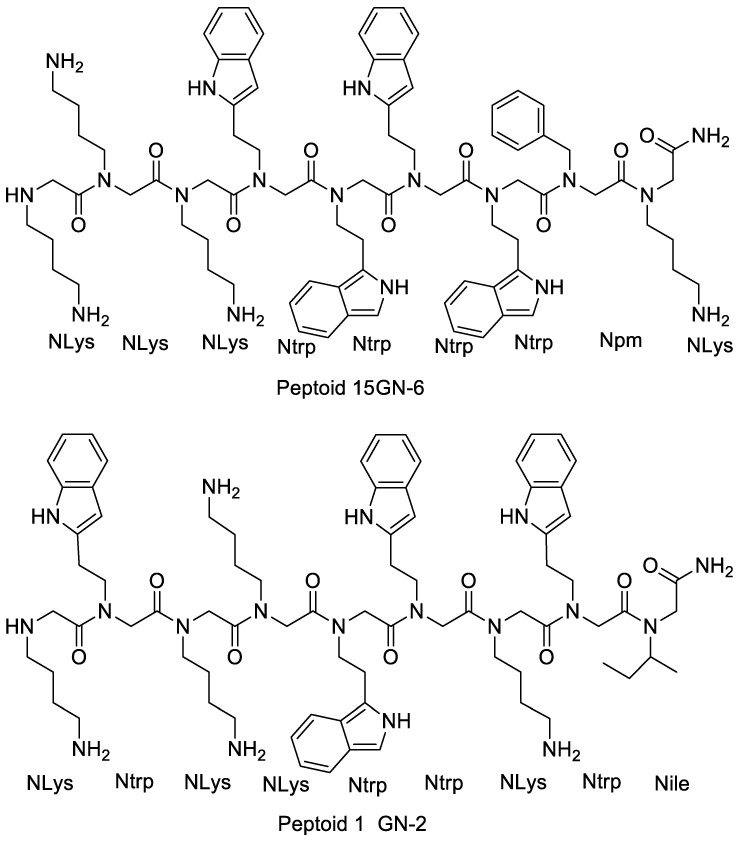
2D structures of peptoid 4, peptoid 15, and peptoid 1.

Several structural analogs where monomers on the peptoid were rearranged to give altered amphiphilicity and charge distribution were used to investigate the influence of this rearrangement on the peptoid’s potency. Mojsoska et al. (2015) found that disarranging the charge cluster at the amine-terminus of peptoid resulted in a more prominent hydrophobic character and no considerable improvement in the activity against *S. aureus* was observed. However, activity increased by four times against Gram-negative strains of *P. aeruginosa* and *E. coli*. Hemolytic activity was not considerably affected by this shift. In contrast to peptoid 15, peptoid 4 (Figure 11) appeared more harmful to HeLa cells. Wimley proposed a model that states that peptides with an imperfect arrangement of hydrophobic and charged residues show increased potency for disrupting bacterial membranes. The potency enhancement against Gram-negative bacteria due to the rearrangement in amphiphilicity for peptoid 4 agrees with this model. Furthermore, disturbing the hydrophobic region in melittin improved the capability to form pores [12].

**Figure 12:**
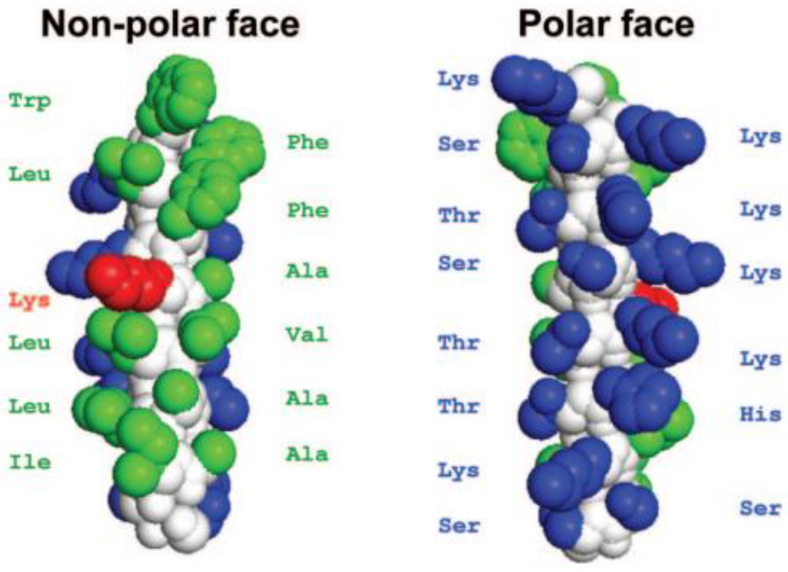
The space-filling model of parent peptide V13K_L_. The region colored in white represents the backbone of the peptide. The amino acids in green color are hydrophobic, while the ones in blue color are hydrophilic. The Lys substitution on the 13th position (V13K_L_) on the non-polar face of the helix is highlighted in red [44]. Reprinted with permission from [44], published by American Society for Microbiology, 2007.

Disrupting the peptide V13K_L_ dimerization (an amphipathic peptide of 26 amino acids long having a hydrophilic lysine residue in the middle of the non-polar face) (Figure 12) by incorporating a charged amino acid (Lys) in aqueous solution resulted in a decrease in toxicity [12]. A plausible explanation for this is that the dimerization allowed for easier access of the peptide to the interface site of the plasma membranes of mammals while avoiding permeating the membrane. In conclusion, sequence rearrangement can yield peptoids with optimal hydrophobicity that display the greatest selectivity [12].

## 6. Cyclization

**Figure 13:**
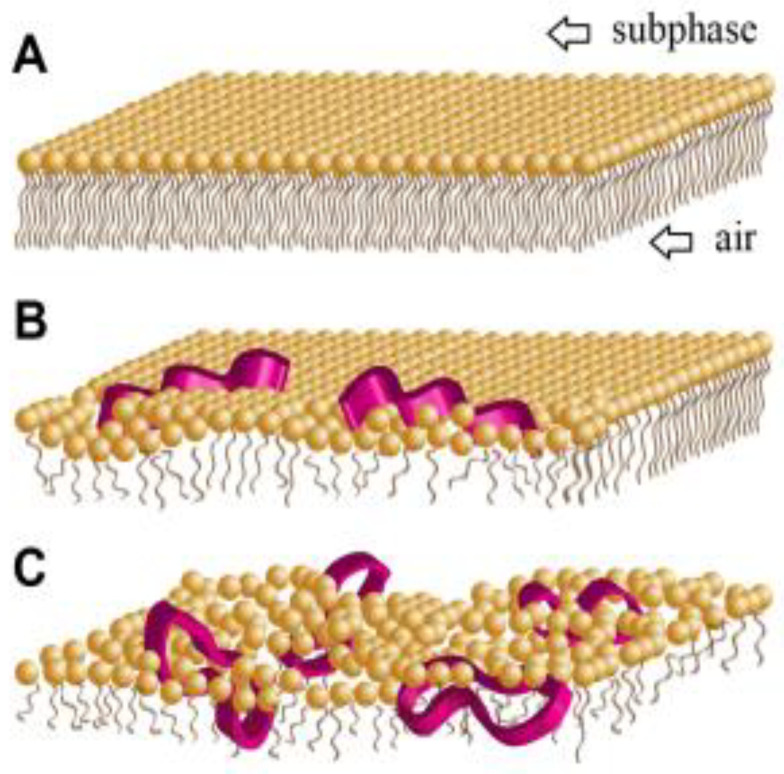
The proposed structural modifications in the outer outlet of the phospholipid membrane of the bacteria (**A**) after inserting linear (**B**) and cyclic (**C**) peptoids. Cyclization enables antimicrobial molecules to intercalate more effectively with lipid film characterization by molecular tilt [52]. Reprinted with permission from [52], published by ACS Publications, 2016.

As stated above, charge, hydrophobicity, amphipathicity, and size are important considerations when designing small membrane-active antimicrobial molecules. The conformational rigidity of these molecules has been recently suggested as an additional vital parameter to consider. Cyclization makes a molecule more rigid without extensive modifications in other physiochemical properties. Cyclic antimicrobial compounds’ reduced structural flexibility might facilitate their ability to penetrate membranes, possibly increasing membrane-disruptive behavior [52]. Unlike linear, flexible molecules, cyclic analogs should experience lower entropy loss during their incorporation into the lipid membrane (Figure 13). Packing disruptions in the lipid matrix frequently result when introducing a molecule into a lipid membrane. The maximization of hydrophobic and electrostatic interconnections between the antimicrobial molecules and the lipids results in the system gaining energy. The accumulative change in energy defines the molecules’ membrane activity [52]. Some reports stated that macrocyclizing antimicrobial peptoids enhanced their membrane activity. Their conformational stability and probable bioavailability make them more attractive as drug candidates.

Fundamental conformational heterogeneity, mainly found in cis–trans amide bond isomerization, is the most difficult aspect when designing peptoids with stable secondary structures. Scientists have come up with several methods to achieve defined conformations in peptoids. Incorporating bulky achiral side chains yields peptoids that take on polyproline type I helix structures, and peptoid sequences with such structures exhibit potent antimicrobial activity. Introducing covalent constraints by head-to-tail macrocyclization is another approach to impose rigidity in peptoid structures. A macrocyclized peptoid has its side chains arranged onto opposite faces of the planar ring. This implies that an amphiphilic peptoid structure could be achieved by properly placing the cationic and hydrophobic side chain groups in the cyclic peptoid sequences. This well-defined amphiphilic structure could result in potent AMP mimetics effective in fighting bacterial pathogens. Studies have previously proven this principle through cyclic peptoid oligomers exhibiting modest antimicrobial effects against bacteria and fungi. When tested against clinically relevant isolates of *S. aureus*, amphiphilic cyclic peptoid oligomers showed strong antibacterial activity. A significant antimicrobial selectivity was observed for these compounds, even though they act on the surface of the bacteria. Furthermore, their non-hemolytic activity and powerful antimicrobial activity are analogous to those recorded for other peptidomimetic oligomers that are presently in clinical development [52].

### Effect of Cyclization on Antimicrobial Activity

**Figure 14:**
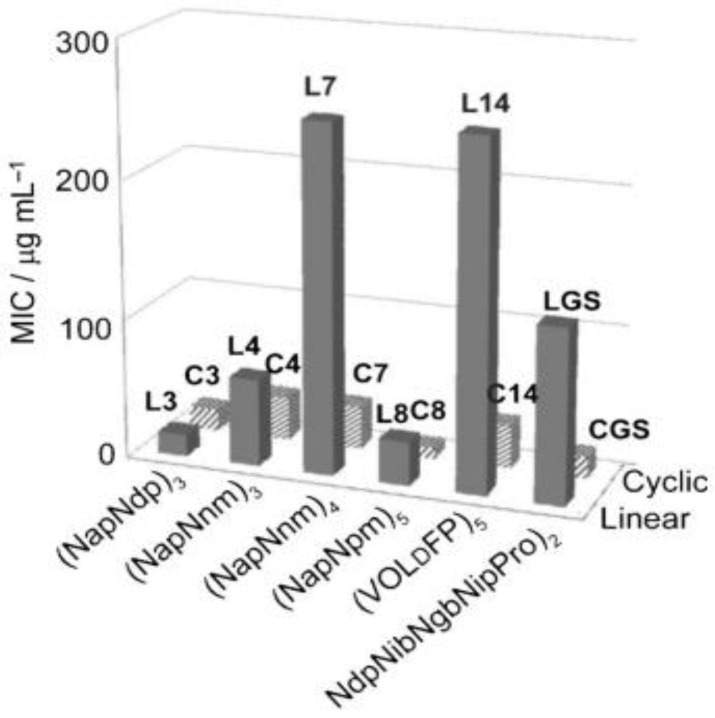
A plot of the six pairs of cyclic (C3, C4, C7, C8, C14, CGS) and linear (L3, L4, L7, L8, L14, LGS) peptoids against the MIC which illustrating the macrocyclized peptoids enhanced the antimicrobial activity against *E. coli* [7]. Reprinted with permission from [7], published by ChemMedChem, 2012.

Several studies demonstrated a compelling connection between the stability of secondary structure brought through macrocyclization and the potential to inhibit bacterial cell growth. The study conducted by Huang et al. focused on Gram-negative (*E-coli*) bacteria to compare the antimicrobial activity of the linear and cyclic peptoid analogs. They observed a decline in MIC values for the cyclic peptoid (Figure 14) analogs correlative to the linear derivatives. This observation led to the conclusion that cyclization generally enhances antimicrobial activity. Among the six pairings of cyclic and linear sequences involved in this study, the cyclic peptoids C7 and C14 showed activity approximately eight-fold more active relative to their linear equivalents (L7 and L14). This improvement in the activity of macrocylized peptoids relative to linear peptoids agrees with the same result acquired by imposing secondary structure in helical peptoid antimicrobial oligomers [7].

## 7. Aromatic Side Chains

### N-aryl Groups within Peptoid Oligomers

**Figure 15:**
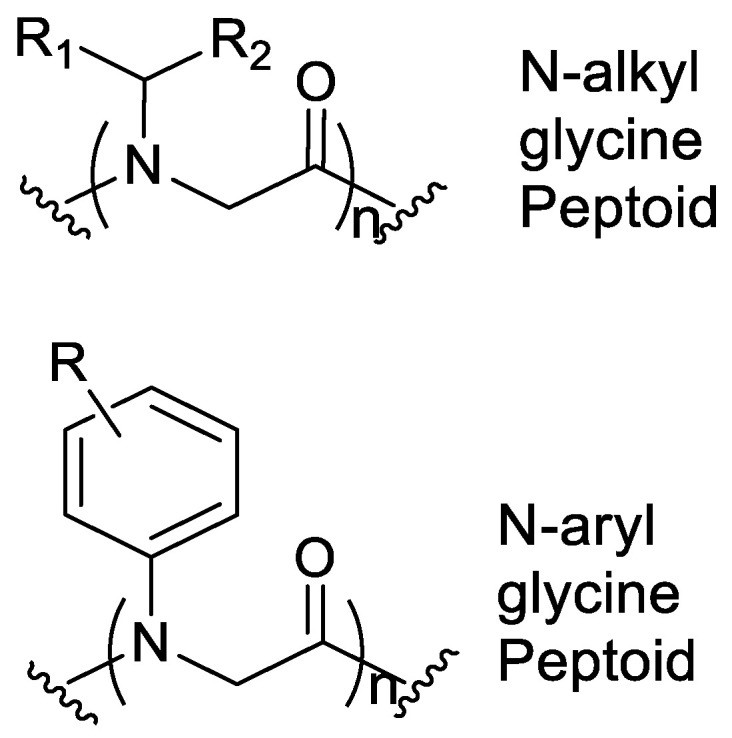
Structural comparison of the *N*-alkyl glycine and the *N*-Aryl Peptoid monomers.

Using N-aryl glycine monomer units (Figure 15) together with other methods might eventually enable the creation of predictable structure-function relationships of peptidomimetic foldamers [53].

Previously, *N*-alkyl glycine monomer units that are relatively flexible have been used in structural investigations of peptoids. Bradley et al. initially proposed that including *N*-aryl side chain groups can potentially lower the conformational heterogeneity. However, no additional investigations were made for this phenomenon. The *N*-aryl glycine monomers can be incorporated to give novel peptoid secondary structures that are conformationally defined in solution. This novel group of peptoid structures might simplify the de novo creation of biomimetic architectures that are chemically diverse [53]. The confined conformations of *N-*aryl glycine oligomers are distinct and encourage backbone conformational stability. 

Including *N*-aryl groups within peptoid oligomers gives them a higher energetic inclination for trans-amide bond geometries. X-ray crystallography and solution NMR spectroscopy studies of *N*-aryl peptoid oligomer structures confirmed that these compounds favor trans-amide bonds across the backbone, and they show the predicted side-chain rotamers. The capability to govern the presence of the trans-amide bonds anywhere within the sequence of the peptoid will improve the ability to foretell the final backbone structure, as indicated in the successful design of *N*-alkyl/*N*-aryl hybrid cyclic hexamers [53].

Regarding the activity of peptoids with N-aryl side chains, one study demonstrated that introducing aromatic residues can lead to the loss of selectivity between the plasmalemma of mammals and bacteria. Besides this, while fine-tuning the hydrophobicity of peptoids, Mojsoska et al. (2015) [12] found that to balance the structural requirements that selectively kill the bacteria, Ntrp and Nai could preferably be utilized as the aromatic monomers.

## 8. Alkylated Peptoids

Alkylated peptides and peptoids are capable of forming micelles at the lowest inhibitory concentration [32,54], which again may increase their local concentration upon their contact with the negatively charged bacterial plasmalemma, if it is presumed that the cationic micelles naturally dissemble succeeding the adsorption to the surface of the membrane. Furthermore, it has been proven that including bulky, branched N-alkyl substituents can create nearby steric interactions that are capable of directing conformational preferences.

### 8.1. Effect of Alkylated Peptoids on Antimicrobial Activity

Fatty acid tails have previously been anchored to linear AMPs that are rarely acylated [55,56], sometimes making cationic peptides that are not active to show antimicrobial activity. The synthesis of peptoids allows for alkylamines to be incorporated within the peptoid as an amine-terminus alkyl tail with ease. Chongsiriwatana et al. (2015) used this method to study alkylated peptoids as mimics of antimicrobial lipopeptides [1]. Following the submonomer strategy, this group created a series of peptoids using suitable alkylamines to incorporate alkyl tails that were 5, 10, or 13 carbons long as side chain groups. They discovered that, in some cases, alkylation remarkably enhanced the selectivity of the peptoids while maintaining antimicrobial potency. Noticeable enhancement in potency was observed for the alkylated peptoids with chain lengths of 9, 6, and 4 residues against bacteria and fungi when contrasted to the peptoids that are not alkylated. 

It has been demonstrated in previous studies that peptoid 1 is active against Gram-positive and Gram-negative bacteria, and the analogs with longer tail lengths and hence more hydrophobic either retain their antibacterial activity or start losing it [28]. However, their antifungal potency increases [28].

**Figure 16:**
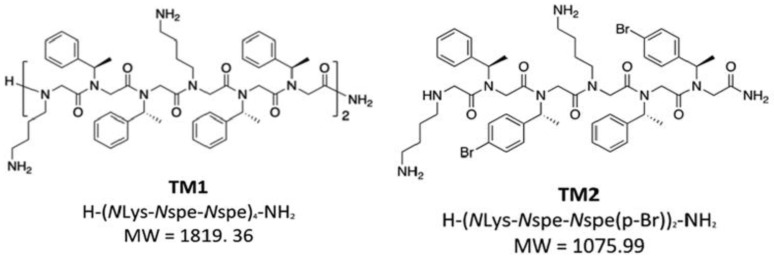

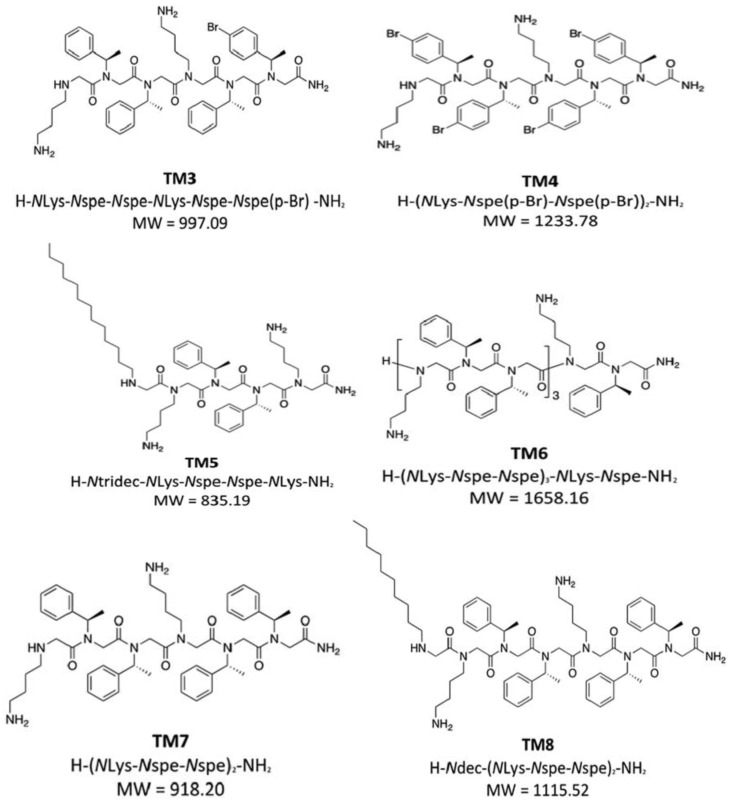

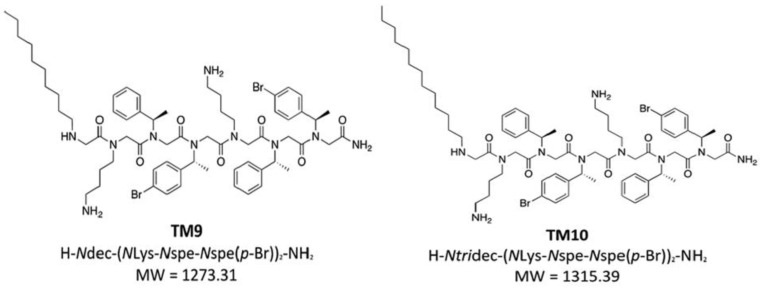
The structures of peptoids TM1 to TM10 [39]. Reprinted with permission from [39], published by ACS Infectious Diseases, 2022.

Nielsen et al. (2022) [39] focused on two well-researched compounds called TM1 and TM5 and eight other variants and molecular hybrids (Figure 16). This group of peptoids differed in their overall positive charge, hydrophobicity, and main chain length (6_mer_–12_mer_) as a result of including various Nspe monomers, halogens, and alkyl chains. 

The analogs TM9 and TM10 are structurally similar and only differ in their alkyl chain length (TM9 has 10 carbons while TM10 has 13 carbons), and it was found that TM10 formed a significant percentage of worm-like micelles. This worm-like morphology was hypothesized to have the ability to inhibit antibacterial and antiviral activity, thus accounting for the reduced activity of TM10 relative to the analogs TM5, TM8, and TM9, which showed antimicrobial activity that is within 2–4 fold of TM1. It can be concluded from these results that micellar aggregation number, as well as hydrophobicity, affect the peptoids’ biological function [39]. 

In agreement with this, a loss in activity was observed for the peptides YGAAKKAAKAAKKAAKAA (AKK) that were conjugated to varying lengths of fatty acids when the minimum active concentration was raised above the critical micelle concentration (CMC). Although this conjugation to fatty acid tails improves their attraction for the negatively charged phospholipid membranes, the self-assembled structure (acquired at concentrations exceeding the CMC) can hinder the effective interaction of the peptide to the plasma membrane of the bacteria [39].

### 8.2. Influence of Alkyl Tail on the Formation of Micellar Structures

Nielsen et al. (2022) reported that adding a terminal alkyl tail to peptoids allows the structures of core-shell micelles to form. These structures have a higher aggregation number when compared to peptoids TM1 and TM6 helical bundles assemble. The formation of ellipsoidal micelle assemblies was observed for peptoids TM5, TM8, and TM9, with aggregation numbers of 98, 103, and 117 peptoids on average, respectively. These remarkable numbers of aggregates propose the existence of intermolecular interactions that exceed hydrophobic forces. These intermolecular interactions could be due to hydrogen bonding between NLys residues and pi-stacking between Nspe residues. The high aggregation numbers also suggest that the physical stability of these ellipsoids is significant, which may be favorable for the successful drug suitability of these peptoids, mainly because these supramolecular peptoid assemblies can function as a vehicle-free self-controlled delivery system. This delivery system further eliminates the need for any physical encapsulation [39].

## 9. Halogen-Substituted Peptoids

According to theoretical modeling, the peptoids TM2 and TM4 from the work of Nielsen et al., work that is substituted with halogens generated bigger helical bundles, possibly as a result of an efficient “hydrophobic” contact between the heavy bromine para-benzyl substituent atoms. TM2, which consists of two Nspe residues that are substituted with bromine atoms, had a 0.005% percentage of bigger aggregates (dimensions of 120 Å × 280 Å × >1000 Å), while for TM4, no larger aggregates were observed. At doses comparable to those of TM1 (1.56 and 12.5 g/mL, respectively), TM2 was able to inhibit the activity of *E. faecium* and *P. aeruginosa*. However, higher concentrations were necessary to prevent the development of the other bacterial species. Likewise, TM4 required lower concentrations (0.78 and 6.25 μg/mL) than TM1 to inhibit *E. faecium* and *P. aeruginosa*, respectively, however, equal or higher concentrations were essential for the inhibition of other bacterial species [39].

## 10. Future Perspectives

Antimicrobial peptoids are synthetic peptides that have shown promise in combating bacterial infections. These peptoids have several advantages over traditional antibiotics, such as increased stability, resistance to degradation, and reduced potential for bacterial resistance. From a future perspective, antimicrobial peptoids can potentially revolutionize the field of medicine. As bacteria evolve and develop antibiotic resistance mechanisms, new treatments are needed to combat these pathogens. Antimicrobial peptoids offer a promising alternative to traditional antibiotics, and ongoing research will likely uncover new and innovative ways to use them. In the coming years, we can expect further development of antimicrobial peptoids, including improvements to their efficacy and safety profiles. This may include the development of new formulations, delivery methods, and combination therapies that can enhance their effectiveness. Additionally, antimicrobial peptoids may also find new applications beyond treating bacterial infections. For example, they may be used to treat fungal infections or cancer, as recent research has suggested that some peptoids have anti-cancer properties. One advantage is that they have a broad spectrum of activity, meaning they can target a wide range of bacteria, including those resistant to traditional antibiotics. This could lead to the development of new treatments for infectious diseases that are currently difficult to treat. Another advantage of antimicrobial peptoids is that they are less prone to resistance development than traditional antibiotics. This is because peptoids have a unique chemical structure, making it difficult for bacterial resistance development. This could help to address the problem of antibiotic resistance, which is a major global health concern. In addition, antimicrobial peptoids have the potential use in various applications beyond the treatment of infections. For example, they could be used in food preservation or development of new materials with antimicrobial properties.

However, there are also challenges associated with the use of antimicrobial peptoids. For example, they can be expensive and difficult to produce on a large scale. There is also a need to develop effective delivery methods to ensure that the peptoids can reach their target site in the body. Another challenge associated with peptoids is the poor peptoid-protein interaction due to the lack of structure on peptoids [57]. Overall, the future of antimicrobial peptoids is bright, and ongoing research in this area will likely yield new and exciting discoveries that could significantly impact human health. Further research and development are needed to fully realize their potential as a new class of antibiotics and antimicrobial agents.

Structure-activity relationship (SAR) studies of antimicrobial peptoids involve understanding how changes in the molecule’s structure affect its activity against different strains of bacteria.

In the future, SAR studies of antimicrobial peptoids will likely continue to play a critical role in developing new antimicrobial agents. With new computational methods and tools, researchers can now model the interactions between peptoids and bacterial cells at the atomic level. These computational techniques can help researchers to design and optimize peptoids with enhanced potency, selectivity, and stability.

Moreover, researchers can also use SAR studies to investigate the mechanisms of action of antimicrobial peptoids. Understanding these ’compounds’ mode of action can help researchers develop new strategies to combat antibiotic resistance, such as combining peptoids with other antibiotics or immune-stimulatory agents. The future of SAR studies of antimicrobial peptoids is bright, and it holds great promise for developing new and effective treatments for drug-resistant bacteria.

## 11. Conclusions

In this review, we outlined recent work on the SAR of antimicrobial peptoids that mimic naturally occurring antimicrobial peptides. While we have evaluated how different structural features like chain length, hydrophobicity, cyclization, net charge, and amphiphilicity each influence antimicrobial activity, it is important to note that the design of peptoids with better antimicrobial, hemolytic, and cytotoxic activity may require a balance between these features. An excellent illustration is the finding that the antibacterial properties of helical peptoids, which are effective against a wide range of microbes, depending on both their total charge and average hydrophobicity and high hydrophobicity and amphipathicity, resulted in hemolysis. Peptoids also showed selective antibacterial action due to their net positive charge and sufficient but moderate hydrophobicity. There is hope that peptoid agents may represent a new and underrepresented class of new antibiotics.

## Figures and Tables

**Table 1:** The retention times of the peptoids and their corresponding in vitro antibacterial activities, hemolytic activities, and cytotoxicity. Reprinted with permission from [12], published by AAC, 2015 [12].

Peptoid No.	Peptoid Nomenclature	Sequence ^f^ (N-C)	*R_t _* (min) ^a^	MIC (µg/mL) for Strain ^b^:						Hemolytic Concn (µg/mL) ^c^	SR ^d^	Cytotoxicity (µg/mL) ^e^
				*S. aureus*		*E. coli*		*P. aeruginosa*				
				ATCC 21213	C623 MRSA	ATCC 25922	63103 ESBL	PAO1	H1027 MDR			
**1**	GN-2	H-*N*lys-*N*trp-*N*lys-*N*lys-*N*trp-*N*trp-*N*lys-*N*trp-*N*ile-NH2	10.54	64	32	32	32	32	4	>128	>2–32	168
**3**	GN-2-*N*lys_1–4_*N*trp_5–8_	H-*N*lys-*N*lys-*N*lys-*N*lys-*N*trp-*N*trp-*N*trp-*N*trp-*N*ile-NH2	11.48	8	8	32	8	32	4	128	4–32	110
**7**	GN-4	H-*N*lys-*N*trp*-N*lys-*N*lys-*N*trp-*N*trp-*N*lys-Ntrp-*N*leu-NH2	10.51	32	ND	64	ND	64	ND	>128	>2–4	166
**9**	GN-4-*N*ai_2,5,6,8_	H-*N*lys-*N*ai-*N*lys-*N*lys-*N*ai-*N*ai-*N*lys-*N*ai-Nleu-NH2	12.31	4	4	16–32	32	16–32	4	32	1–8	172
**21**	Nlys_1–4_*N*trp_5–8_	H-*N*lys-*N*lys-*N*lys-*N*lys-*N*trp-*N*trp-*N*trp-*N*trp-NH2	11.06	4	ND	16	ND	ND	ND	64	4–16	ND

^a^ The estimated analytical retention durations (Rt) were performed using a reverse-phase C18 Kinetex 100 by 2.1 mm 100-column at 60 °C for 20 min with a gradient of 15 to 65% acetonitrile. ^b^ Median MICs, which represent 3 to 5 replicates, are reported in g/mL. ^c^ The results of three separate trials employing several blood donors were used to calculate hemolysis. ^d^ Selectivity ratio (SR) is the ratio of the bacterial strains’ lowest and highest MIC values to 10% hemolysis. ^e^ Cytotoxicity is presented as the IC_50_, which refers to the concentration that inhibits 50% of the metabolic activity of HeLa WT cells utilizing the colorimetric tetrazolium salt-based MTS assay. IC_50_ for peptides as determined by an MTT experiment on HeLa WT cells. Antimicrobial peptides are taken as reference antimicrobial compounds. ^f^ Submonomeric solid-phase peptoid synthesis was used to make peptoids [12].

**Table 2:** The linear and cyclic peptoids with their corresponding antimicrobial and hemolytic activities [7].

	Sequence ^a^	MIC [µg·mL^−1^] ^b^			HC [µg·mL^−1^] ^c^		SR ^d^
		*B. sub.*	*E. coli*	*S. aur.*	HC_50_	HC_10_	
**L3**	Ac(NapNdp)_3_	1	15.6	15.6	>250	>62.5	>4
**L4**	Ac(NapNnm)_3_	2	62.5	125	>250	>250	>4
**L6**	Ac(NapNpm)_3_	125	125	250	>250	>250	>2
**C3**	C(NapNnm)_3_	1	15.6	7.8	>250	31.3	>2
**C4**	C(NapNnm)_3_	0.5	31.3	31.3	>250	>250	>8
**C6**	C(NapNpm)_3_	500	>500	500	>250	>250	NA

^a^ The prefixes Ac and C, respectively, are utilized to refer to N-acetylated linear sequences. ^b^ Minimum inhibitory concentrations (MICs) against *Bacillus subtilis*, *Escherichia coli*, and *Staphylococcus aureus*. ^c^ Hemolytic concentrations: HC_50_, concentration at which 50% hemolysis is observed; HC_10_, concentration at which 10% hemolysis is observed. ^d^ Selectivity ratio: SR = HC_10_/MIC *E. coli*; the abbreviation NA stands for not applicable because there was insufficient antibacterial activity; three independent trials were done in three replicates [7].

## Data Availability

Not applicable.
